# Integrating bulk and single-cell sequencing reveals the phenotype-associated cell subpopulations in sepsis-induced acute lung injury

**DOI:** 10.3389/fimmu.2022.981784

**Published:** 2022-11-02

**Authors:** Fuquan Wang, Ming Chen, Jiamin Ma, Chenchen Wang, Jingxu Wang, Haifa Xia, Dingyu Zhang, Shanglong Yao

**Affiliations:** ^1^ Department of Anesthesiology, Union Hospital, Tongji Medical College, Huazhong University of Science and Technology, Wuhan, China; ^2^ Department of Anesthesiology, Institute of Anesthesia and Critical Care Medicine, Union Hospital, Tongji Medical College, Huazhong University of Science and Technology, Wuhan, China; ^3^ Wuhan Jinyintan Hospital, Tongji Medical College, Huazhong University of Science and Technology, Wuhan, China

**Keywords:** sepsis, lung injury, single-cell sequencing, Scissors-method, cellular landscape

## Abstract

The dysfunctional immune response and multiple organ injury in sepsis is a recurrent theme impacting prognosis and mortality, while the lung is the first organ invaded by sepsis. To systematically elucidate the transcriptomic changes in the main constituent cells of sepsis-injured lung tissue, we applied single-cell RNA sequencing to the lung tissue samples from septic and control mice and created a comprehensive cellular landscape with 25044 cells, including 11317 immune and 13727 non-immune cells. Sepsis alters the composition of all cellular compartments, particularly neutrophils, monocytes, T cells, endothelial, and fibroblasts populations. Our study firstly provides a single-cell view of cellular changes in septic lung injury. Furthermore, by integrating bulk sequencing data and single-cell data with the Scissors-method, we identified the cell subpopulations that are most associated with septic lung injury phenotype. The phenotypic-related cell subpopulations identified by Scissors-method were consistent with the cell subpopulations with significant composition changes. The function analysis of the differentially expressed genes (DEGs) and the cell-cell interaction analysis further reveal the important role of these phenotype-related subpopulations in septic lung injury. Our research provides a rich resource for understanding cellular changes and provides insights into the contributions of specific cell types to the biological processes that take place during sepsis-induced lung injury.

## Introduction

Sepsis is an organ dysfunction characterized by high morbidity, severity, and mortality due to the dysregulated host response to infection (1), which imposes a huge burden on human health and socioeconomics (2). Although considerable research has been devoted to the pathogenesis and treatment of sepsis, the exact pathogenesis of sepsis has not been elucidated, and the clinical outcomes of sepsis patients are still unsatisfactory. The lung is the first and most commonly damaged organ in sepsis (3). Approximately 50% of patients with sepsis develop acute lung injury (ALI) (4). ALI and its more severe form, acute respiratory distress syndrome(ARDS), are the leading cause of death in sepsis patients (5).

Sepsis induced-ALI is characterized by the overactivated inflammatory response, disruption of the alveolar-capillary membrane barrier, pulmonary edema, and pulmonary dysfunction. Numerous immune cells and non-immune cells are involved in the occurrence and progression of septic lung injury. Therefore, although research contributing to the pathological mechanism of septic lung injury has been extensively studied for decades, there is currently a lack of effective enough understanding of septic lung injury due to the complex nature and the involvement of multiple cell types (6).

Sepsis-induced lung injury comprises a complex microenvironment of interacting lung structural constituent cells and immune cells. However, the current cellular-level studies on the pathological mechanism of septic lung injury are relatively independent, most of the studies only focus on one or two of the lung cell components such as epithelium, endothelium, monocytes, and neutrophils (3, 7–9), and lack a systematic explanation of the overall cellular changes during lung injury. Studies of septic lung injury that focus only on individual cell types are not enough. In addition, the current research on septic lung injury mainly uses peripheral blood or bronchoalveolar lavage fluid, and there is no systematic elucidation of the damage to the main constituent cells of lung tissue. Therefore, there is an urgent need to elucidate the comprehensive landscape and heterogeneity of immune and non-immune cells of the lung tissue during septic lung injury.

**Figure 1:**
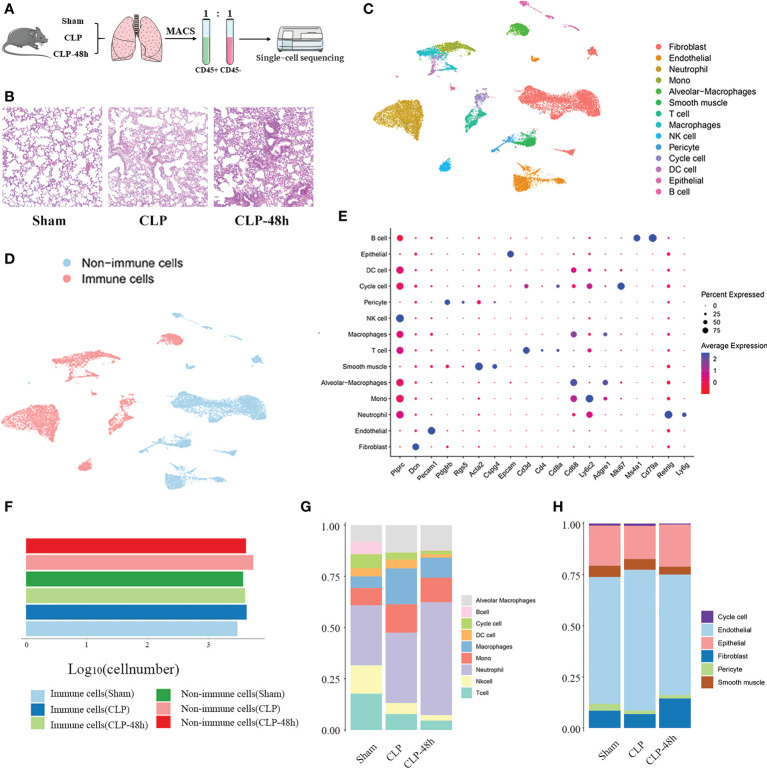
Identifying the infiltrated cell types in septic and non-septic lung tissue. **(A)** The main workflow of the study. **(B)** The He staining results of the lung tissue (magnification 200×). **(C)** The UMAP plot of all scRNA-seq data, shows a total of 14 distinct cell types that were identified. **(D)** UMAP plot of sequenced cells grouped by color by immune and non-immune cells. **(E)** Dot plot for cell-type-specific signature genes. **(F)** The number of immune cells and non-immune cells in each group. **(G)** The proportion of various types of cells in different groups of immune cells. **(H)** The proportion of various types of cells in different groups of non-immune cells.

Single-cell RNA sequencing (scRNA-seq) is an efficient tool for exploring cell heterogeneity (10). Therefore, by using the well-established mouse model of sepsis-induced lung injury, we performed single-cell sequencing on mouse lung tissue to develop a deeper and more accurate understanding of the changes and interactions of various cell types in the lung tissue microenvironment during septic lung injury (The main flow of the experiment is shown in **Figure 1A**). The single-cell RNA sequencing is capable of distinguishing cell types, states, and lineages well. However, the current single-cell data cannot directly link cell clusters to specific phenotypes. The Scissors-method developed by Xia Zheng et al. can identify cell subsets related to a given phenotype by integrating phenotype-associated bulk expression data and single-cell data (11). So we also performed bulk RNA sequencing and used the Scissors-method to identify the cell subpopulations most associated with septic lung injury.

Here, we report extensive profiling of the cellular composition in the sepic mouse lung by generating scRNA-seq profiles of 25044 cells. We provide a detailed cell atlas of normal and septic mouse lung tissue. We also identified the cell subpopulations closely associated with the septic lung injury phenotype with Scissors-method and revealed the specific genes, gene function enrichment, and interactions with other cell clusters for these subpopulations. Our results provide insights into sepsis-associated lung injury and provide an essential resource for the future discovery of effective treatment strategies.

## Methods

### Ethics statement

The experiment was performed in accordance with the Chinese Animal Research Guidelines and was approved by the Laboratory Animal Management Committee of Tongji Medical College of Huazhong University of Science and Technology.

### Animals

Six weeks old male C57BL/6 mice (weight, 20–25 g) were housed in the standard laboratory at 22°C with 40–60% and 12 h/12 day/night cycle. The mice had free access to food and water. The mice were allowed to adapt to the environment 1 week before the experiment. The mice were randomly assigned to three groups: sham group, sepsis group (including CLP group, and CLP-48h group).

We used the Cecal ligation and puncture (CLP), the most commonly used method to induce polymicrobial sepsis, to construct the mouse model of septic lung injury. CLP was performed as described previously (12). The mice in the Sham group and CLP group were sacrificed 24 hours after the operation, while the CLP-48h group was sacrificed 48 hours after the operation. The lung tissue samples were collected for subsequent testing and analysis. We performed bulk RNA sequencing on 3 Sham group specimens and 3 CLP group specimens. Three sham group specimens, three CLP specimens, and three CLP-48h specimens were mixed for single-cell RNA sequencing.

### Hematoxylin and eosin staining

Lung tissue was fixed with 4% paraformaldehyde and embedded in paraffin. The paraffin was then cut into 5 µm sections, deparaffinized, rehydrated, and stained with H&E. The pathological changes in lung tissue were observed under a light microscope.

### RNA sequencing

The total lung RNAs were used for RNA-seq. The RNA sequencing libraries were constructed and sequenced on the BGISEQ-500 platforms.

### Single-cell suspension preparation

After being isolated from the mice, the fresh lung tissue was rinsed twice with the pre-cooled RPMI 1640 + 0.04% BSA medium under sterile conditions. Then the samples were sufficiently cut into small pieces of about 0.5 mm^3^. Then the samples were incubated with a mixture of 2 ml of trypsin (Gibco, Cat: R001100), 1 ml of collagenase II (Biofrox, Cat:2275MG100), and 100 µl of DNase (Servicebio, Cat: 1121MG010) for 1 h on a 37°C shaker. The digested cell suspension was filtered 1-2 times with a BD 40 μm cell sieve and centrifuged at 300 g for 5 min at 4°C. After the pellet was resuspended with an appropriate amount of medium, an equal volume of red blood cell lysate (MACS, Cat. No. 130-094-183) was added. After mixing, let stand at 4°C for 10 min, the cell suspension was centrifuged at 300g for 5 min, and the supernatant was discarded. The pellet was washed once with medium and centrifuged at 300 g for 5 min, the supernatant was discarded, the cell pellet was resuspended with 100 μl of the medium, and the cell concentration and viability were calculated by Luna cell counter.

For CD45 sorting, the prepared single-cell suspensions were subjected to Magnetic-activated cell sorting (MACS) using Miltenyi mouse CD45 magnetic beads according to the manufacturer’s recommendations. The CD45- and CD45+ cell suspensions were mixed 1:1 for single-cell sequencing.

After adjusting the cell concentration of the single-cell suspension to 700-1200 cells/μl, the single-cell library was prepared following the protocol of the 10×Genomics Chromium Next GEM Single Cell 3′ Reagent Kits v3.1. High-throughput sequencing was performed on the constructed library using the Illumina Nova 6000 PE150 platform.

### Single-cell data preprocessing

Cell Ranger (version 5.0.0) was used to process the raw data and generate the raw unique molecular identifier(UMI) count matrix. The merged matrix was transferred into the R statistical environment for further analysis using the Seurat package (v. 4.1.2).

The cells with UMI counts < 1000, mitochondrial UMI counts greater than 10%, or below 500 genes were identified as low-quality cells and removed (**Supplementary Figure 1A**, All Supplementary Figures are in the “Supplementary Figures” file). The gene expression matrix was normalized using the “NormalizeData” function and 2000 highly variable genes (HVGs) were identified by the “FindVariableFeatures” function. Batch effects were corrected by the “Harmony” function. Principal component analysis (PCA) was performed to reduce the dimension of the scRNA-Seq dataset. By the “ElbowPlot” function in Seurat, we used TOP 50 PCs for downstream analysis (**Supplementary Figure 1B**).

The cells were clustered and identified by the “FindNeighbors” and “FindClusters” functions with a resolution of 1. The clusters were projected into a two-dimensional plot for visualization using the “RunTSNE” and “RunUMAP” functions.

### Cluster marker identification and cell-type annotation

The differentially expressed genes(DEGs) of each cluster were identified by the “FindAllMarkers” function in Seurat, the clusters were annotated according to the expression of classic markers. When identifying the main cell types, the cells that expressed more than one typical cell type marker were considered double cells and excluded from further analysis. Firstly, all cells were divided into 14 major cell types, and then the major cell types were further clustered and annotated separately to detect heterogeneity within each cell type.

### “Scissor” method identifies the cell subpopulations associated with sepsis-induced lung injury

“Scissor” integrates the single-cell data and phenotype-relevant bulk sequencing data by first quantifying the similarity between each bulk and each cell sample. The scissor method can then optimize the regression model on the matrix associated with the sample phenotype to identify relevant subpopulations. A detailed description can be found in the literature (11).

### Pseudotime analysis

The “Monocle 3” package (13) was used to infer the potential lineage differentiation trajectory.

### Functional enrichment analysis

GO enrichment of DEGs of cell subgroups was performed using the “compareCluster” function in the “clusterProfiler” packages (14).

### Cell-cell communication analysis

CellPhoneDB2, a Python-based computational analysis tool developed by Roser Vento-Tormo et al. (15), was used to analyze cell-to-cell communication at the molecular level.

## Results

### Detailed map of cellular composition in the sepsis-induced lung injury

The condition of the lung tissue of the mice was evaluated by HE staining and the results showed that the mice in the CLP group and the CLP-48h group had obvious lung injury (**Figure 1B**). To create a comprehensive cellular map of the normal and septic lung, we first isolated and sequenced a total of 31532 cells from lung tissue suspensions derived from three groups, including the Sham group (8742 cells), CLP group (12159 cells), CLP-48h group (10631 cells).

After standard data processing and the low-quality cells were filtered out, we performed downstream analysis on a total of 25044 lung cells, including 6825 cells from the Sham group, 9914 cells from the CLP group, and 8305 cells from the CLP-8h group. Based on the expression of the classic markers genes (**Figure 1E**; **Supplementary Figure 1C**), we clustered and annotated these cells into 14 major cell types (**Figure 1C**), including Cycle cell, Alveolar Macrophages, Mono cell, Macrophages, DC cell, neutrophil cell, B cell, NK cell, T cell, endothelial cell, epithelial cell, Fibroblast cell, smooth muscle cell, pericyte cell.

To reveal the cellular composition of normal lungs and septic lungs, we first divided the cells into immune cells and non-immune cells according to the expression of CD45 (**Figures 1D, F**; **Supplementary Figure 1C**), and the results showed that the numbers of immune cells and non-immune cells were in the same order of magnitude, indicating that magnetic bead sorting was effective.

To reveal the cellular composition of immune and non-immune cells, we compared the relative percentages of each cell type. Among immune cells (**Figure 1G**), neutrophil, mono, and T cells were the most three prevalent subsets, while in the non-immune cell fraction (**Figure 1H**), endothelial cells and fibroblast cells are relatively dominant. Next, we mainly characterized the transcriptomic features of the main cell clusters.

### The heterogeneity analysis of monocytes

**Figure 2:**
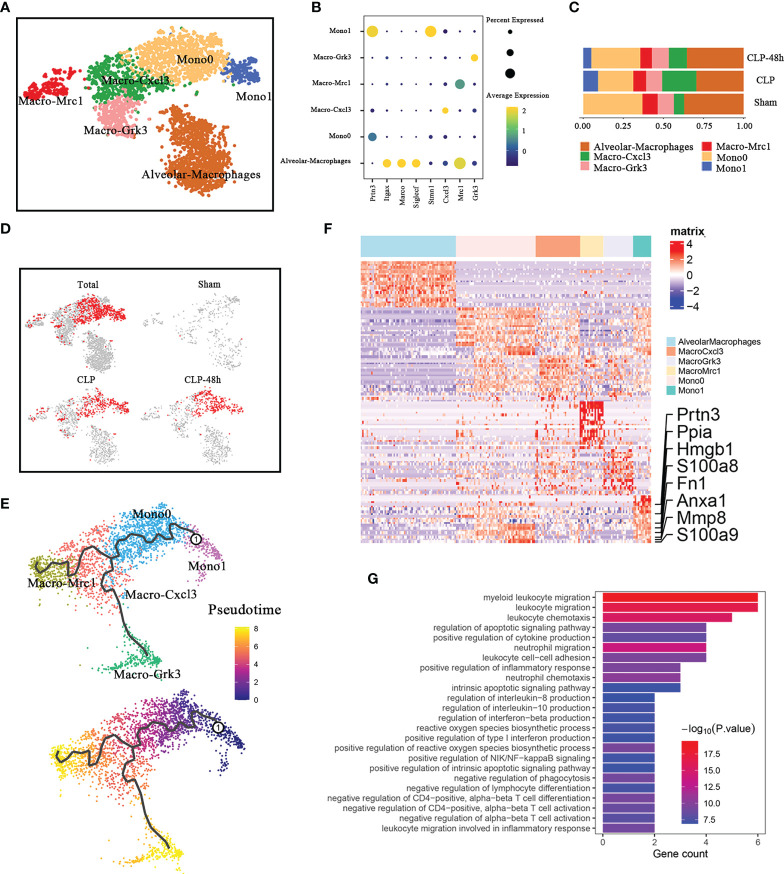
Cellular composition of monocytes in normal and septic lung tissue. **(A)** a total of five clusters of monocytes were identified in lung tissue. Cell subpopulations are colored as shown in the legend. **(B)** Dot plot for principal identifiers of different subclusters. **(C)** The proportions of different monocyte subclusters. **(D)** The UMAP visualization of the Scissor-medthod selected cells. The red dots are cells associated with the sepsis phenotypes. **(E)** The prediction of monocyte differentiation trajectories with Monocle. **(F)** Heatmap of DEGs of the Mono1 subcluster. **(G)** Enriched GO functions of upregulated genes in Mono1.

Monocytes represent the first line of defense against infection. In the early stages of infection and sepsis, monocytes undergo activation, and functional and morphological changes following infectious stimulation, showing a high degree of heterogeneity. Based on the differences in marker genes (**Figure 2B**), we clustered the monocytes into six subgroups (**Figure 2A**), including Alveolar Macrophages, Mono0, MacroCxcl3, MacroMrc1, MacroGrk3, and Mono1.

We observed that Mono1 was specifically identified in septic lung injury, the proportion of which in the CLP group was higher than that in the CLP-48h group (**Figure 2C**). The scissors method suggested that Mono1 cluster were the subclusters that were most associated with the sepsis-induced lung injury phenotype (**Figure 2D**). Of note, the percentage of Mono0 was decreased in the CLP group compared with the Sham group, which was even lower than the CLP-48h group, while the proportion of Macro-Cxcl3 showed an obvious opposite trend. To determine if there is a link between Mono0 and Mono1, we performed the pseudotime analysis. The Monocle3 pseudotime analysis suggested (**Figure 2E**) that Mono0 was differentiated from Mono1 clusters. The mono1 population may be a specific cell cluster migrating from peripheral blood under the condition of septic lung injury. Interestingly, the proportion of Mono1 in the CLP-48h group is lower than that in the CLP group, indicating that Mono1 may be closely related to the acute phase of sepsis-induced lung injury. We further identified the DEGs of Mono1, as expected, the DEGs of Mono1 were shown to possess the characteristics of promoting inflammatory response, including S100A8/A9, Hmgb1, Mmp8, etc. (**Figure 2F**). The Go enrichment analyses of the Mono1 DEGs were predominantly involved in the inflammatory signals, including chemotaxis of neutrophils and lymphocytes, regulation of cytokine production, etc. (**Figure 2G**). Therefore, Mono1 is more likely to be the most important subgroup that aims to promote the inflammatory response in sepsis acute lung injury.

Among the DEGs, the most noteworthy gene is Hmgb1 and S100A8/A9. A considerable number of studies have been published to confirm the important roles of S100A8/A9 in inflammatory-related diseases (16, 17). Previous studies have indicated that the upregulation of S100A8/A9 monocytes may contribute to cytokine storms in severe patients (18). Our study shows that S100A8/A9+ monocytes are also closely associated with septic lung injury. It is worth noting that the expression level of S100A8/A9 in the CLP-48h group was higher than that in the CLP group, and began to be significantly expressed in the Mono0 subgroup (**Supplementary Figure 2A**).

As a ubiquitous nuclear protein that can be secreted by activated monocytes/macrophages, the role of HMGB1 in orchestrating inflammatory responses has been demonstrated in previous studies (19, 20). Furthermore, clinical evidence suggests that the levels of circulating Hmgb1 are positively associated with severity and mortality in septic patients (21). Compared with S100a8/a9, the expression of Hmgb1 showed more obvious subgroup specificity, our study showed that in septic lung injury, Hmgb1 is not indiscriminately elevated in all monocytes/macrophage populations, but is concentrated in the Mono1 subclusters, especially the Mono1 subpopulation in the CLP group.

We next investigated the network of cell-cell interactions among Mono1 and other immune cells identified in our current work by using CellphoneDB2.0 (**Supplementary Figure 2B**). Notably, CellphoneDB is only applicable to human genes, and it is necessary to convert mouse genes into human genes for relevant analysis. Mono1 expresses higher levels of CCR1, the receptors of which includes CCC15, CCL18, and CCL23 (The corresponding relationships in cellphone are: CCL15-Ccl6, CCL18-Ccl3, CCL23-Ccl6). Interestingly, CCC15, CCL18, and CCL23 of Mono1 cells were also highly expressed. In addition, the above-mentioned Ccr1 receptor-ligand pair is mainly expressed in some subsets of monocytes/macrophages and neutrophils. This suggests that the chemotaxis of Mono1 is regulated by other immune cells and that Mono1 is also responsible for the infiltration of certain other immune cells.

### The heterogeneity analysis of neutrophils

**Figure 3:**
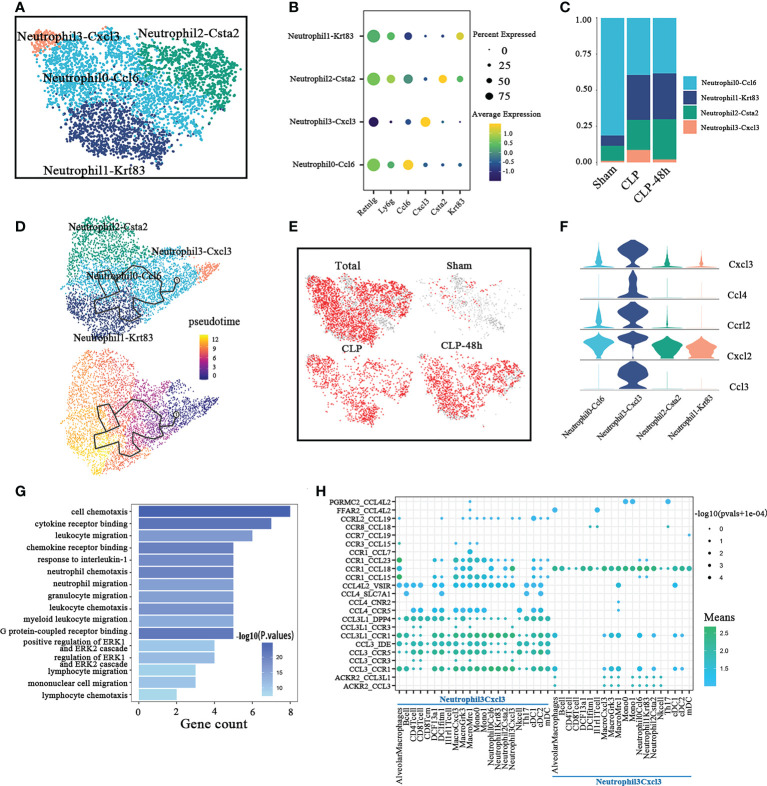
Cellular composition of neutrophils in normal and septic lung tissue. **(A)** a total of four subclusters of neutrophils were identified in lung tissue. Cell subpopulations are colored as shown in the legend. **(B)** Dot plot for principal identifiers of different subclusters. **(C)** The proportions of different monocyte subclusters. **(D)** The prediction of neutrophils differentiation trajectories with Monocle. **(E)** The UMAP visualization of the Scissor-method selected cells. The red dots are cells associated with the sepsis phenotype. **(F)** Violin plots of DEGs for Neutrophil3-Cxcl3. **(G)** Enriched GO functions of upregulated genes in Neutrophil3-Cxcl3. **(H)** Dot plot show ligand-receptor pairs of cytokines between Neutrophil3-Cxcl3 and other immune cell groups.

Excessive accumulation and activation of neutrophils in lung tissue is the main pathological feature of septic lung injury (22). Previous studies have suggested that neutrophil populations are not homogenous (23). We identified four distinct neutrophil clusters (**Figure 3A**) based on their expression profiles (**Figure 3B**), including Neutrophil0-Ccl6, Neutrophil1-Krt83, Neutrophil2-Csta2, Neutrophil3-Cxcl3. The analysis of the composition of neutrophils in each group showed that the neutrophils in sepsis-related lung tissue were significantly different from the normal lung tissue (**Figures 3C, D**). Among them, the proportion of Neutrophil3-Cxcl3 subclusters in the CLP group was significantly increased.

To demonstrate the developmental progression among different neutrophil clusters described above, we ordered these clusters by performing the trajectory analysis using Monocle 3 (**Figure 3E**). Mme is an important marker of neutrophil maturation. So we also analyzed the expression of Mme in different neutrophil subpopulations. The results showed that although the overall expression level was very low, the expression level of Mme in the Neutrophil3-Cxcl3 subpopulation was still significantly lower than that of other subgroups(As shown in **Supplementary Figure 3A**). Our results suggested that the Neutrophil3-Cxcl3 subpopulation is more likely to be derived from peripheral blood, then differentiate into Neutrophil0-Ccl6, and then differentiate into Neutrophil1-Krt83 and Neutrophil2-Csta2. The scissors method suggested that almost all neutrophils in the CLP and CLP-48h groups were associated with the phenotype of septic lung injury. Combining the above findings, we consider the Neutrophil3-Cxcl3 subclusters to be the main driver of lung injury in the CLP group.

The highly expressed genes in Neutrophil3-Cxcl3 (**Figure 3F**), such as Cxcl2, Cxcl3, and Ccl3, have been reported to involve in neutrophil and other immune cell migration (24–26). The GO analyses (**Figure 3G**) also suggested the DEGs in Neutrophil3-Cxcl3 are mainly enriched in the chemotaxis and migration of neutrophils, lymphocytes, monocytes, etc. So we next explored the cell-cell interaction network between Neutrophil3-Cxcl3 and other immune cell types(Figure). CellphoneDB results (**Figure 3H**) showed that Neutrophil3-Cxcl3 mainly expressed CCL18, the receptor of which was mainly expressed on certain neutrophils, monocytes/macrophages, and DC cell subsets. Neutrophil3-Cxcl3 also highly expresses the chemokine receptor Ccr1, the ligand of which is expressed in a variety of immune cells, indicating that the migration and infiltration of C during septic lung injury is regulated by a variety of immune cells. Our studies suggested that the chemotaxis of Neutrophil3-Cxcl3 may play a positive feedback effect on inflammatory cell recruitment. Furthermore, the major chemokines and chemokine receptors of Neutrophil3-Cxcl3, such as Ccl3, and Cxcr1, may be potential main intervention targets for inhibiting neutrophil-sustained inflammatory conditions in septic lung injury.

### The heterogeneity analysis of lymphocytes

**Figure 4:**
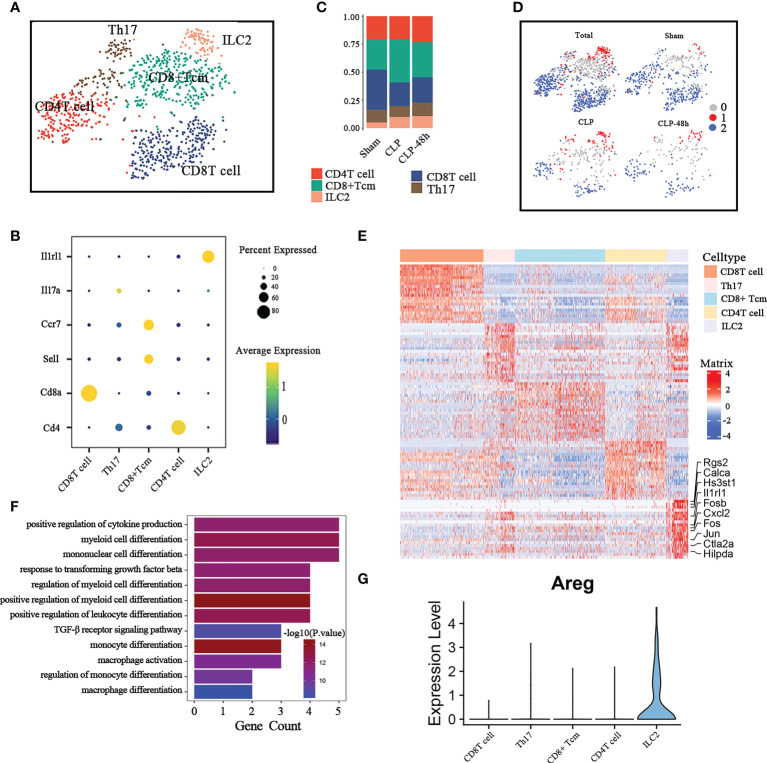
Cellular composition of lymphocytes in normal and septic lung tissue. **(A)** a total of five subclusters of lymphocytes were identified in lung tissue. Cell subpopulations are colored as shown in the legend. **(B)** Dot plot for principal identifiers of different subclusters. **(C)** The proportions of different lymphocyte subclusters. **(D)** The UMAP visualization of the Scissor-method selected cells. The red and blue dots are cells associated with sepsis and normal phenotypes **(E)** Heatmap of DEGs of the ILC2 subcluster. **(F)** Enriched GO functions of upregulated genes in ILC2. **(G)** Violin plot of the expression levels of Areg in different lymphocyte subclusters.

We identified 5 subgroups of T cells based on the expression of classic T cell markers (**Figure 4B**), including “CD8 Tcell”, “Th17 cell”, “CD8 TCM”, “CD4 Tcell”, and “ILC2” (**Figure 4A**). We originally selected T lymphocytes for further analysis, but during the subpopulation annotation process, we found a subcluster that negatively expressed Cd3d, Cd4, and CD8a, but was positive for Gata3(**Supplementary Figure 4A**), which was identified as ILC2.

We observed that the proportion of CD8 Tcell was significantly reduced and the ILC2 cell was significantly increased in the sepsis group compared with the Sham group (**Figure 4C**). The scissors method suggested that ILC2 was the subgroup most associated with the sepsis phenotype (**Figure 4D**), so we focus on the ILC2 subclusters for further analysis. Considering that ILC2 is mainly expressed in the CLP group, we directly compared the DEGs between ILC2 and other subclusters. The top10 differential genes are shown in **Figure 4E**.

Previous studies have shown that ILC2 plays an important role in regulating adaptive immunity, macrophage polarization, etc (27, 28). The results of the Go enrichment analysis (**Figure 4F**) showed that DEGs of ILC2 were mainly focused on regulating innate immune cells, including regulating the myeloid cell differentiation, and positive regulation of cytokine (18). As a member of the epidermal growth factor (EGF)family, amphiregulin (Areg) has been implicated in exerting an important role in regulating tissue repair in acute epithelial injury and asthma (29, 30). ILC2 is an important source of Areg in the lung (31), however, the expression of Areg in ILC2 is significantly inhibited in septic lung injury (**Figure 4G**; **Supplementary Figure 4B**), which may be one of the important mechanisms of septic lung injury.

### The heterogeneity analysis of endothelial cells

**Figure 5:**
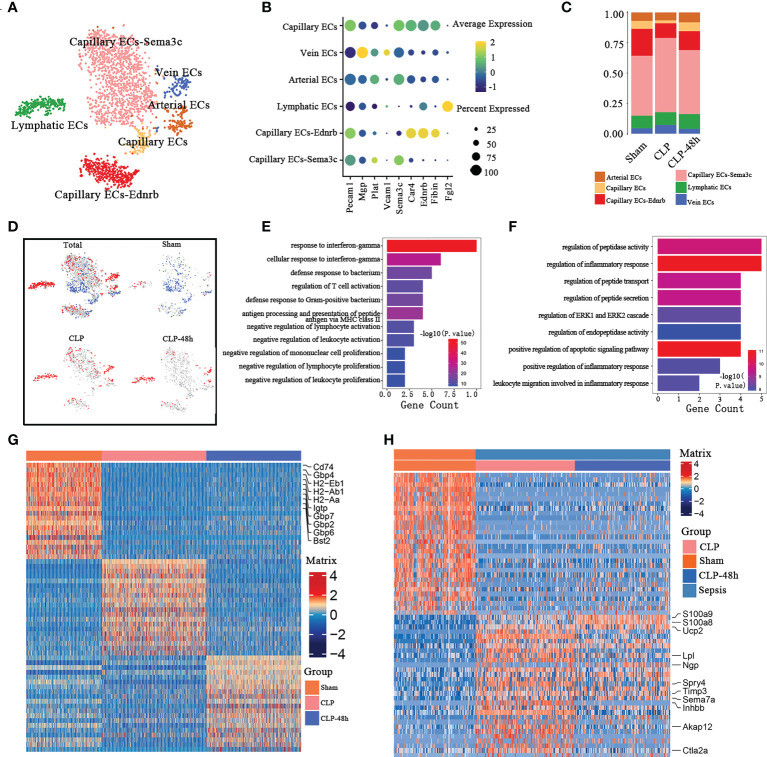
Cellular composition of endothelial cells in normal and septic lung tissue **(A)** a total of six subclusters of endothelial cells were identified in lung tissue. Cell subpopulations are colored as shown in the legend. **(B)** Dot plot for principal identifiers of different subclusters. **(C)** The proportions of different lymphocyte subclusters. **(D)** The UMAP visualization of the Scissor-method selected cells. The red and blue dots are cells associated with sepsis and normal phenotypes. **(E)** Enriched GO functions upregulated genes of Capillary ECs-Sema3c and Capillary Ecs in the Sham group. **(F)** Enriched GO functions upregulated genes of lymphatic ECs in the sepsis group. **(G)** Heatmap of DEGs of Capillary ECs-Sema3c and Capillary ECs subpopulations in the Sham group. **(H)** Heatmap of DEGs of lymphatic ECs in the sepsis group.

Inflammation and dysfunction of endothelial function play a central role in sepsis-induced lung injury. We identified endothelial cells (ECs) into six clusters of distinct expression profiles (**Figure 5B**), including Capillary ECs-Sema3c, Capillary ECs-Ednrb, lymphatic ECs, Arterial ECs, Vein ECs, Capillary ECs (**Figure 5A**).

The percentage of endothelial cell composition in each group showed the cell subsets with the most obvious percentage changes were Capillary ECs-Ednrb and Capillary ECs cells (**Figure 5C**). The scissors method showed that populations of Capillary ECs-Sema3c and Capillary Ecs are the workhorses of endothelial cells against septic lung injury, while the lymphatic ECs are closely related to the progression of sepsis(**Figure 5D**). So we selected the three subclusters mentioned above for further analysis. The top10 DEGs of the Capillary ECs-Sema3c and Capillary ECs of the Sham group are shown in **Figure 5G**. We found that the DEGs of Capillary ECs-Sema3c and Capillary ECs in the Sham group were mainly enriched in the function of suppressing inflammatory responses, including in the response to interferon γ, promoting the activation of T cells and inhibiting other immune cells (**Figure 5E**). Immune interferon-gamma (IFN-γ) plays an important role in fighting infection and exerts its effects through an extensive transcriptional program involving approximately 2000 genes (32, 33), of which the Guanylate-binding proteins (GBP) family is an important member. GBPs are a family of dynamin-related large GTPases which are expressed in response to interferons. The antibacterial function of GBPs has been confirmed by multiple studies (34, 35), consistent with the GO enrichment showing that the Sham group had an increased response to interferon-γ. The gene expression of GBP2, GBP6, and GBP7 in the Sham group was significantly higher than that in the sepsis group.

CD74, the major histocompatibility complex II-associated invariant chain, mainly expresses antigen-presenting cells (APC), which is related to the function of antigen presentation. Studies have also found that CD74 molecules are also expressed in endothelial cells (36) which are related to the occurrence and development of corresponding diseases (37). The expression of CD74 was significantly decreased in the sepsis-associated group, which might be associated with the excessive inflammatory response (38). Moreover, CD74 is an indispensable part of the receptor complex of macrophage migration inhibitory factor (MIF), and macrophages can produce IL-8 that chemotactic leukocytes to the site of infection (39). The expression of CD74 in the sepsis-related group was significantly inhibited, which may be one of the reasons for the excessive activation of inflammatory cells.

The scissor method showed that the ECs clusters most associated with the septic lung injury phenotype were lymphatic ECs. GO enrichment analysis of lymphatic ECs showed that the DECs (**Figure 5H**), were enriched in the regulation of inflammatory response and regulatory peptide synthesis, transport, secretion, and other related pathways (**Figure 5F**). Previous studies have shown that endothelial cells can be involved in sepsis progression by regulating the expression of related peptides, which is consistent with our findings.

Our study also found that lymphatic ECs DEGs were associated with the migration of granulocytes, so we next explored the cell-cell interactions of lymphatic ECs and other immune cells in the CLP group. CellphoneDB results (**Supplementary Figure 5A**) showed that the lymphatic ECs of the CLP group mainly expressed Ccl7 (**Supplementary Figure 5B**), the receptor of which includes Ccr1 and Ccr2, thereby exerting chemotactic effects on certain subsets of neutrophils and monocytes. Notably, there were significant interactions in the CLP group between lymphatic ECs and the subpopulations that are closely associated with the septic lung injury phenotype, such as Mono1, and Neutrophil-Cxcl3.

### The heterogeneity analysis of fibroblasts

**Figure 6:**
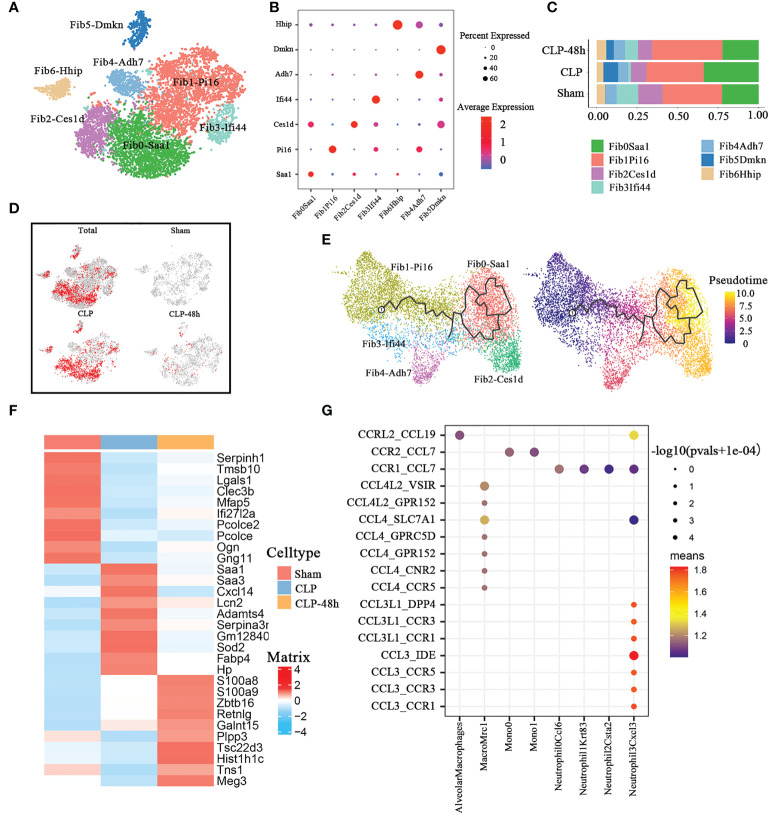
Cellular composition of fibroblasts in normal and septic lung tissue. **(A)** a total of seven subclusters of fibroblasts were identified in lung tissue. Cell subpopulations are colored as shown in the legend. **(B)** Dot plot for principal identifiers of different subclusters. **(C)** The proportions of different fibroblasts subclusters. **(D)** The UMAP visualization of the Scissor-method selected cells. The red and blue dots are cells associated with sepsis and normal phenotypes. **(E)** The prediction of fibroblast differentiation trajectories with Monocle. **(F)** Heatmap of top10 DEGs of different groups of fibroblasts. **(G)** Dot plot show ligand-receptor pairs of cytokines between fibroblasts and other immune cell groups in the CLP group.

In the current study, our studies reveal the diversity of fibroblast cells with seven cell clusters (**Figure 6A**) based on differences in marker genes (**Figure 6B**). There were significant differences in the fibrocystic constituents of the lung tissue of normal mice and septic mice (**Figure 6C**).

The Scissors-method showed that most of the fibroblasts in the CLP group were not innocent of septic lung injury, especially the constituent cells of the Fib0-Saa1 and Fib2-Ces1d subpopulations (**Figure 6D**). In addition, the Fib5-Dmkn cluster was only present in the sepsis-related group. The results of Monocle3 pseudotime analysis (**Figure 6E**) showed that Fib0-Saa1 and Fib2Ces1d subpopulations are differentiated from Fib1-Pi16.

We directly performed differential gene analysis on the fibroblasts of the three groups, and the top 10 differential genes were shown in **Figure 6F**. The results of the Go enrichment analysis (**Supplementary Figure 6A**) showed that the differential genes of fibroblasts in the CLP group were mainly concentrated in the acute inflammatory response, lipoprotein-related pathways, and reactive oxygen pathways. The Serum-Amyloid A(SAA) family protein genes SAA1 and SAA3 were the main players involved in the GO pathway. As one of the typical positive acute phase response proteins, SAA is closely related to infectious diseases. In the process of inflammation, plasma concentrations of SAA (mainly SAA1) increase dramatically by up to 1,000-fold (40). SAA could serve as a major diagnostic marker in the acute phase of inflammation in animals and humans. The chemotactic properties of SAA protein for monocytes and neutrophils have been confirmed (41, 42). We found that the expression of SAA1 and SAA3 was significantly increased in the CLP group, especially in the Fib0-Saa1 and Fib2-Ces1d subpopulations(**Supplementary Figure 6B**).

Previous studies have shown that damage-responsive fibroblasts can alter the lung microenvironment to promote robust infiltration of immune cells at the expense of lung function (43). So we analyzed the interaction between fibroblasts and immune cells in the CLP group. CellphoneDB results (**Figure 6G**) showed that the fibroblast of the CLP group mainly expressed Ccl7 (**Supplementary Figure 6C**). Notably, fibroblasts in the CLP group, similar to lymphatic ECs, both exhibited significant chemotaxis to cell subclusters associated with the septic lung injury phenotype, including Mono1 and Neutrophil-Cxcl3.

## Discussion

Despite intense research efforts aimed at exploring the pathogenesis and treatment of sepsis-induced lung injury, there is still a lack of effective means to address this clinical challenge. Surviving severe sepsis-induced lung injury depends on a careful balance between building an immune response sufficient to clear the infection and maintaining lung function in the presence of immune-induced tissue damage. There is an urgent need to clarify the precise pathogenic mechanisms of sepsis-induced ALI to identify new therapeutic targets.

The host inflammatory response dominated by immune cells can effectively limit the spread of pathogens and eventually clear the pathogens, but immunopathology is also a major factor in tissue damage and ARDS (5, 44). Non-immune lung cells, including fibroblasts, endothelial cells, and epithelial cells are also involved in coordinating immune responses and are directly involved in the regulation of lung function (45). Therefore, the coordination of the functions of immune cells and non-immune cells of the system is the key to solving the lung injury caused by sepsis. However, there is currently no systematic study of immune and non-immune cells of the lung cell lineages in the context of sepsis-induced lung injury. Therefore, we depicted the high-resolution visualization of the cellular landscape in the septic lung *via* scRNA-seq technology, which will facilitate a better understanding of the pathogenesis of sepsis-induced lung injury.

We firstly provide extensive profiling of cellular composition in sepsis-induced lung injury by using multiplexed scRNA-seq to assess the expression profile of 25044 cells. Monocytes, neutrophils, T cells, endothelial cells, and fibroblasts were then selected as major cellular components for further analysis. Our study provides insight into the pathogenesis of sepsis-induced lung injury by characterizing several pathological cell subpopulations with distinct molecular expression profiles.

We used the Scissors-method to identify the cell subpopulations most associated with the septic lung injury phenotype. During the analysis of the major subpopulations, we found that the most phenotypically correlated cell subclusters identified by the scissors method were consistent with the subclusters, the proportions of which varied significantly across the three groups. This also proves to a certain extent that the cell subsets we focus on are closely related to septic lung injury.

It is generally believed that inflammatory storm is an important pathological mechanism of acute lung injury in sepsis, and innate immune cells play a major role in this process. As the first line of defense against the invasion of foreign microorganisms, neutrophils were once considered to be the same type of terminally differentiated white blood cells, with single functional characteristics, short lifespan, and low transcriptional activity. However, recent studies have shown that neutrophils have phenotypic and functional heterogeneity under different physiological or pathological conditions (46). Neutrophils and monocytes are key components of the innate immune system, the excessive activation of which is known to cause organ dysfunction in sepsis (47). However, our study shows that not all immune cells are murderers. In the case of monocytes, the Scissors-method suggested that only the Mono0 and Mono1 subclusters were strongly associated with the septic lung injury phenotype. While in the neutrophils, nearly all of them showed phenotype-related transcriptional changes during septic lung injury.

Many past studies have attempted to address acute lung injury from sepsis by focusing on attenuating epithelial and endothelial cell damage (22, 48, 49). However, not all non-immune cells, including epithelial and endothelial cells, which are more often considered “victim” roles, are innocent during septic lung injury. Previous studies suggested that the physiological function of endothelial cells is severely disrupted during sepsis, and not only functions as a physical barrier (50, 51) but actively participates in the regulation of physiological and pathophysiological processes (52). Our study shows that in the process of septic lung injury, mainly lymphatic endothelium plays a negative regulatory role in an endothelial cell population, and in fibroblasts, mainly Fib0-Saa1 and Fib2-Ces1d are involved in the lung injury process. These non-immune cell subsets also cannot be ignored in the exploration of septic lung injury.

No single subpopulation can function alone during septic lung injury, so we also performed cellular communication analysis for subpopulations with significant heterogeneity, primarily those associated with the lung injury phenotype. Our results suggested that cell subclusters more associated with the septic lung injury phenotype, such as neutrophil3-Cxcl3 and Mono1 subclusters, are more strongly associated with endothelial cells, fibroblasts, and many other immune cells. These cells not only expressed high levels of chemokine receptors but also highly expressed chemokine ligands, which may be associated with the generation of inflammatory storms.

Our study also revealed many possible therapeutic targets, such as our study showed that ILC2 highly expressed Areg, while the expression of Areg of ILC2 in the CLP group was significantly inhibited, which may be an intervention target for promoting airway epithelial integrity and lung tissue homeostasis. For another example, both lymphocytes and endothelial cells associated with the phenotype of septic lung injury highly express the chemokine Ccl7, which plays a chemotactic role for some phenotype-related immune cells. This suggested that Ccl7 in non-immune cells may also be a clue for the intervention of septic lung injury.

There are several limitations in our study, for example, the total number of cells involved in our study is not very large, resulting in the annotation of some cell subclusters may not be detailed enough. Furthermore, because our study was more focused on the comprehensive landscape of immune and non-immune cells in lung tissue during septic lung injury, mainly at 24 h, deep functional validation of individual subpopulations was lacking. In our opinion, the effective functional verification of the identified subpopulation most related to the sepsis lung injury phenotype is to use gene knockout animals, preferably lung tissue conditional knockout mice. Knock out specific functional genes of relevant subsets respectively to explore whether the symptoms and prognosis of sepsis-induced lung injury can be improved. We will continue to explore relevant work in depth in the future. Nevertheless, our study may provide foundations for further exploration of the mechanism of septic lung injury to a certain extent.

In conclusion, our study is the first to systematically provide a cellular transcriptional landscape of lung tissue in sepsis-induced lung injury. We also identified the subpopulations of cells most associated with the septic lung injury phenotype and revealed the transcriptional signature of these subpopulations. Our study will advance a better understanding of the pathogenic roles of different lung cell clusters during sepsis-induced lung injury.

## Data availability statement

The original contributions presented in the study are publicly available. This data can be found here: https://www.ncbi.nlm.nih.gov/geo/query/acc.cgi?acc=GSE207651.

## Ethics statement

The animal study was reviewed and approved by Institutional Animal Care and Use Committee of Huazhong University of Science and Technology(IACUC Number:2629).

## Author contributions

FW, MC, and JW established the animal models and collected samples. FW, JM, and CW finished the bioinformatics analysis. JW and HX prepared the figures. FW wrote this article. SY. and DZ designed the experiments and revised the article. All authors contributed to the article and approved the submitted version.

## Funding

The study was supported by the Major Technological Innovation Special Project of Hubei Province of China (2019ACA167).

## Acknowledgments

We would like to thank OE Biotech Co., Ltd (Shanghai, China) for providing single-cell RNA-seq, Dr. Junyi Hu (Department of Urology, Tongji Hospital, Tongji Medical College, Huazhong University of Science and Technology) for the guidance of bioinformatics analysis.

## Conflict of interest

The authors declare that the research was conducted in the absence of any commercial or financial relationships that could be construed as a potential conflict of interest.

## Publisher’s note

All claims expressed in this article are solely those of the authors and do not necessarily represent those of their affiliated organizations, or those of the publisher, the editors and the reviewers. Any product that may be evaluated in this article, or claim that may be made by its manufacturer, is not guaranteed or endorsed by the publisher.
