# Supplementary material for: Integrating bulk and single-cell sequencing reveals the phenotype-associated cell subpopulations in sepsis-induced acute lung injury
Source: Front Immunol. 2022 Nov 2;13:981784. doi: 10.3389/fimmu.2022.981784 (PMC9666384; doi:10.3389/fimmu.2022.981784)

Supplementary Figures:

Supplementary Figure 1 Quality control of single-cell sequencing data A: Vlnplot shows the number of genes (nFeature_RNA), number of UMI (nCount_RNA), detected and percent of mitochondrial-derived transcripts (percent. mt) per single cell after quality control. B: Scree plot shows the top50 PCs of principle component analysis. Top50 PCs were used in downstream analysis. **C.** The UMAP visualization of the expression of marker genes for major cell types.

Supplementary Figure 2

A: tSNE plot of the expression of S100a8/a9 and Hmgb1 in different subclusters of monocytes. **B.** Dot plot show ligand-receptor pairs of cytokines between Mono1 and other immune cell groups.

Supplementary Figure 3

A: The Dot plot of the expression level of Mme in different neutrophil subpopulations.

Supplementary Figure 4

A: tSNE plot of the expression of Cd3d, Cd4, Cd8a, and Gata3 in different types of lymphocytes. B: Violin plot of the expression levels of Areg in different groups of ILC2

Supplementary Figure 5

A. Dot plot show ligand-receptor pairs of cytokines between lymphatic ECs and other immune cell groups in the CLP group. **B.** Violin plot of the expression levels of Ccl7 in different groups of lymphatic ECs.

Supplementary Figure 6

A: Enriched GO functions of the DEGs of the fibroblasts in the CLP group. **B:** tSNE plot of the expression of Saa1 and Saa3 in different subclusters of fibroblasts.**C:** Violin plot of the expression levels of Ccl7 in different groups of lymphatic ECs

Supplementary Figure 1

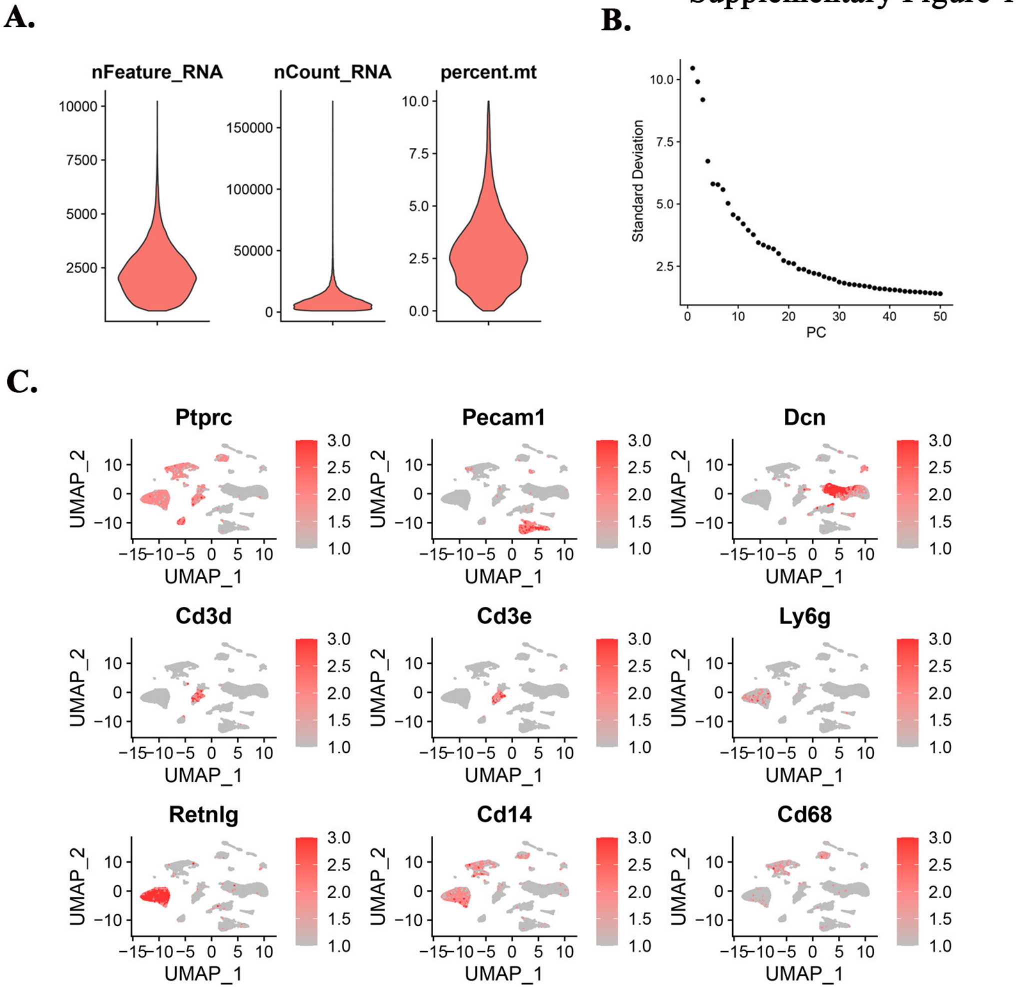

Supplementary Figure 2

A.

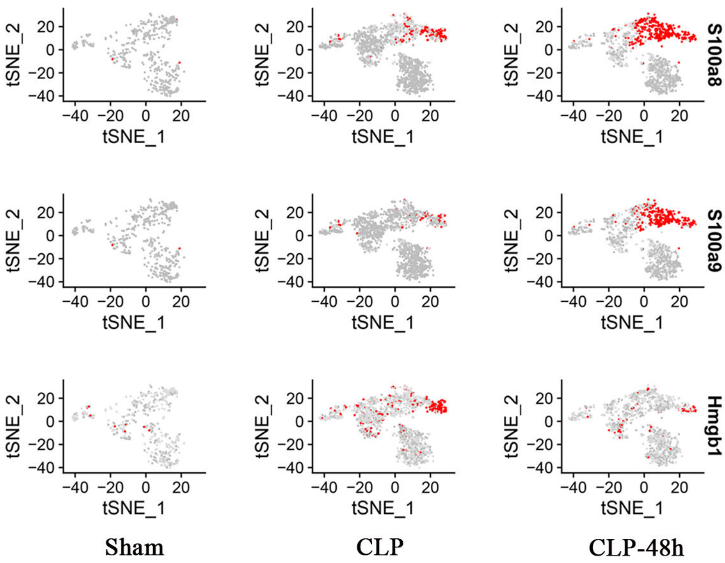

B.

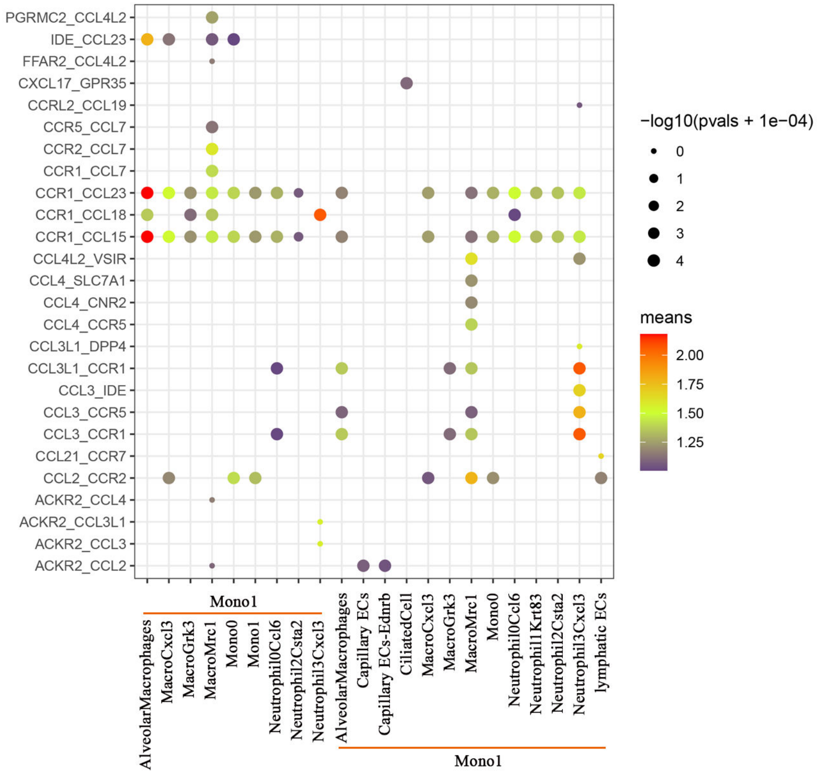

Supplementary Figure 3

A.

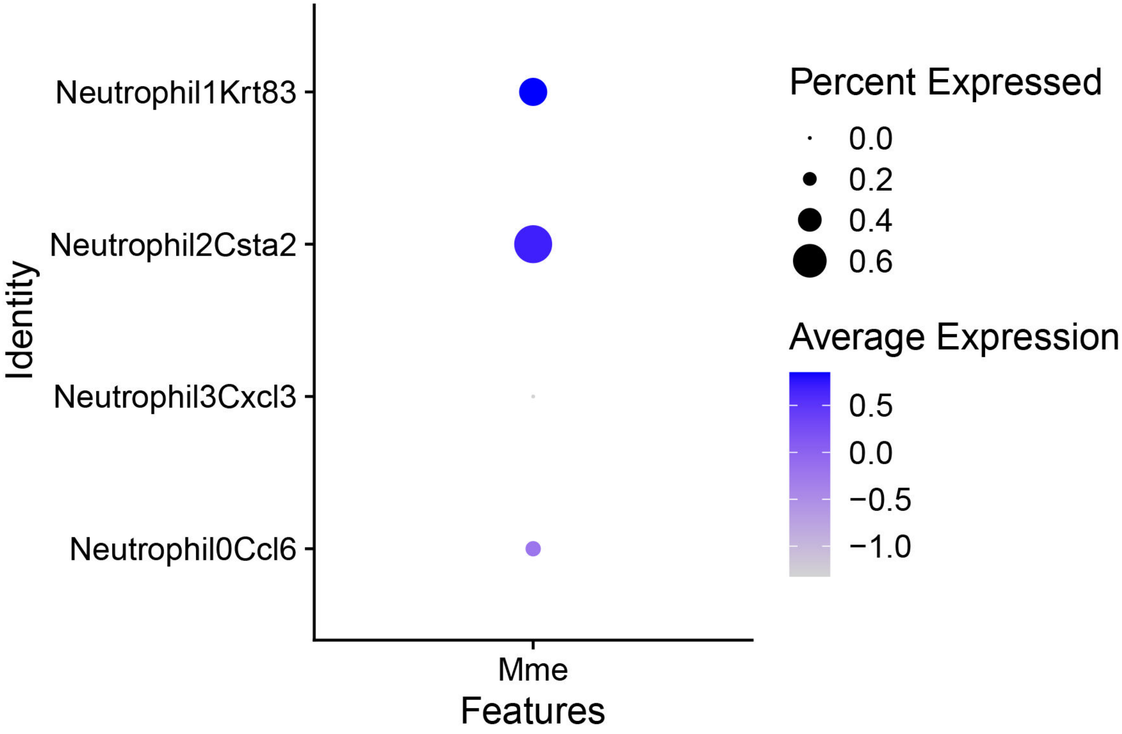

Supplementary Figure 4

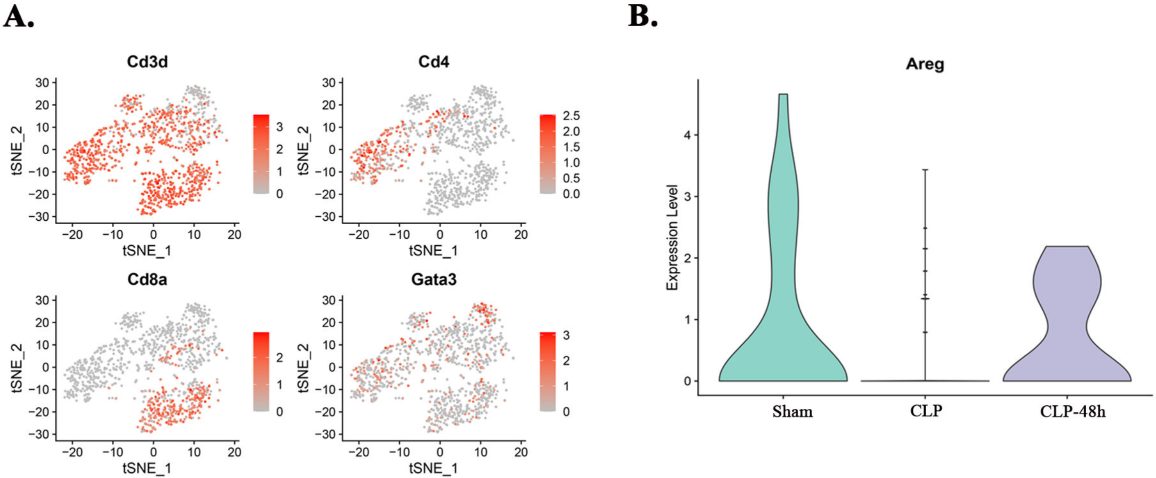

Supplementary Figure 5

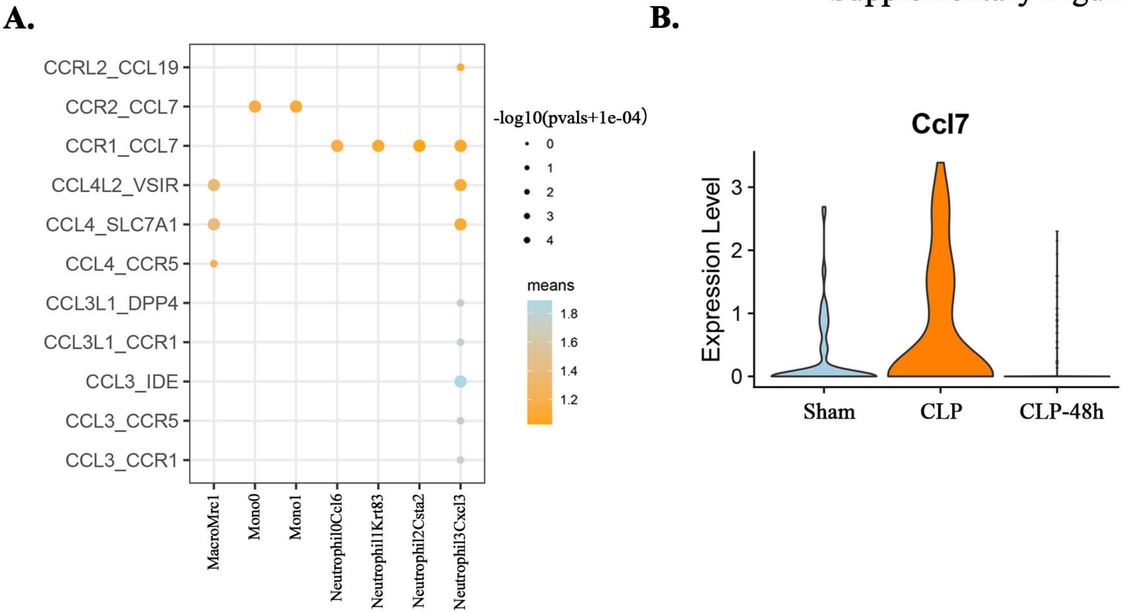

Supplementary Figure 6

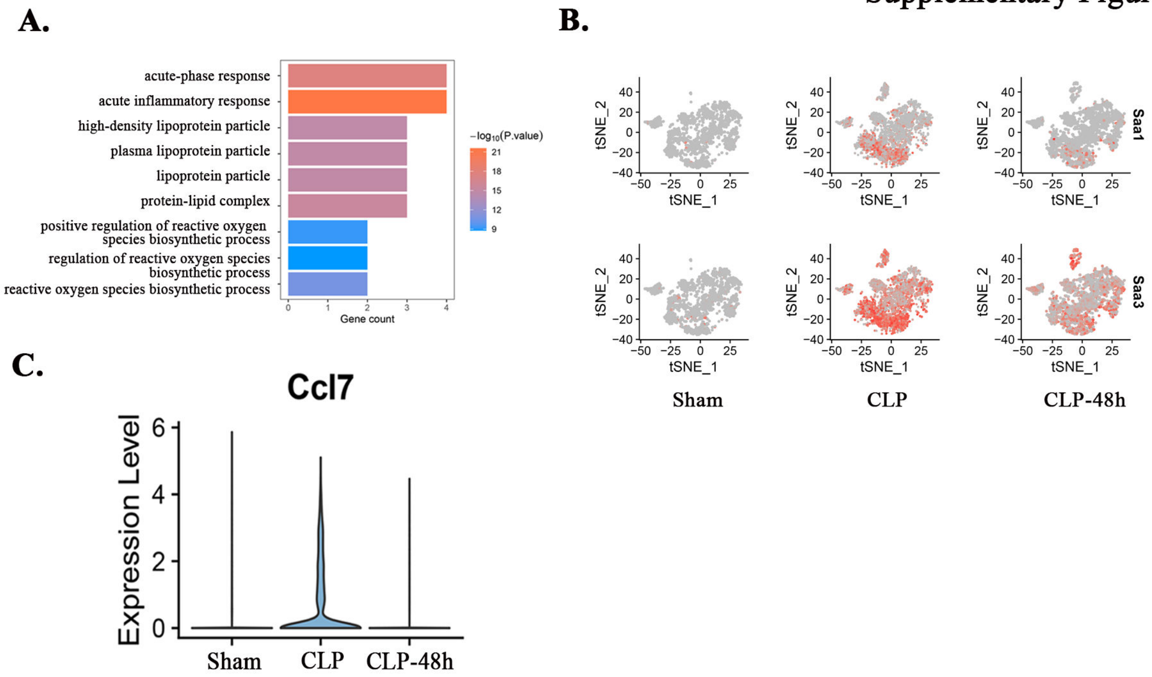

Supplement: Supplementary file 2 [file DataSheet_2.pdf]
